# The Impact of an Internet-Based Self-Management Intervention (HeLP-Diabetes) on the Psychological Well-Being of Adults with Type 2 Diabetes: A Mixed-Method Cohort Study

**DOI:** 10.1155/2016/1476384

**Published:** 2015-11-22

**Authors:** Megan Hofmann, Charlotte Dack, Chris Barker, Elizabeth Murray

**Affiliations:** ^1^Department of Clinical, Educational and Health Psychology, University College London, Gower Street, London WC1E 6BT, UK; ^2^Psychology Department, University of Bath, Claverton Down, Bath BA2 7AY, UK; ^3^Research Department of Primary Care and Population Health, University College London, Upper Floor 3, Royal Free Hospital, Rowland Hill Street, London NW3 2PF, UK

## Abstract

This mixed-method study assessed the impact of an internet-based, self-management intervention (“HeLP-Diabetes”) on the psychological well-being of adults with type 2 diabetes. Nineteen participants were recruited from 3 general practices. Data were collected at baseline and at 6 weeks follow-up. Access to HeLP-Diabetes was associated with a significant decrease in participants' diabetes-related distress (*Z* = 2.04, *p* = 0.04, and *d* = 0.28). No significant differences were found in emotional distress or self-efficacy. The qualitative data found that participants reported improvements including increased self-efficacy and support, better management of low mood, greater diabetes awareness, and taking the condition more seriously. Participants also reported making improvements to their eating habits, exercise routine, and medical management. Some negative experiences associated with using the intervention were mentioned including feelings of guilt for not using the intervention as suggested or not making any behavioral changes, as well as technical and navigational frustrations with the intervention. Internet-based self-management interventions may have the potential to decrease diabetes-related distress in people with type 2 diabetes. The qualitative data also suggests internet interventions can positively impact both psychological and behavioural outcomes of adults with type 2 diabetes.

## 1. Introduction

Type 2 diabetes is a complex and challenging condition affecting around 2.5 million people in the UK [1]. People with diabetes are more likely to develop heart disease, kidney failure, and blindness and to die prematurely than people without diabetes [2]. The risk of developing many of these problems can be reduced if people with type 2 diabetes are given the knowledge and skills to self-manage their condition. This involves making substantial behavioural and lifestyle changes [3]. However the psychological burden of living with diabetes can create significant barriers to managing these demands and achieving treatment goals [4, 5]. If a person is overwhelmed by the changes that need to be made, one response may be to deny the condition, which may lead to a reduction in knowledge, awareness, and skills needed to manage their diabetes [6]. Numerous studies have found that the prevalence of psychological difficulties such as anxiety and eating disorders is higher in people with diabetes than the general population [7, 8]. The prevalence of depression is approximately twice as high [9]. Poor psychological well-being in people with diabetes is associated with suboptimal glycaemic control and increased risk of complications [8, 10]. It is also associated with lower medication adherence, greater difficulties managing medical care, and lost productivity [11]. These findings highlight the importance of improving psychological wellbeing in people with type 2 diabetes in order to facilitate diabetes self-management [12, 13].

Structured education is known to promote self-management and reduce the incidence of diabetes complications [14] as well as improving emotional well-being, quality of life, and diabetes self-efficacy and clinical outcomes such as glycaemic control [15–17]. Examples of existing group-based self-management programmes for people with type 2 diabetes in the UK include DESMOND, X-PERT, and Co-Creating Health. Although these programmes have shown initial benefits [18, 19], there are concerns that benefits may not be sustained in the long term [20]. There are additional concerns that group-based programmes such as these may not suit all patients who need self-management training. People who work, have caring responsibilities at home, who have mobility problems, or who find group interactions difficult may all have difficulty attending. Thus there is an urgent needed to find cost-effective and acceptable methods of delivering sustainable self-management education for people with type 2 diabetes in the UK. In addition, although in 2008 the National Institute of Health and Clinical Excellence (NICE) advised that a key priority for implementation was the offer of structured education to every patient and/or their carer at the time of diagnosis with diabetes with annual reinforcement [21], only a minority of patients report being offered (less than 9%) or receiving (less than 4%) such education [22]. This suggests that health care providers are encountering difficulties implementing and resourcing quality education programmes.

One possible solution to this problem and a potentially alternative and acceptable means of delivering cost effective self-management education is the use of internet-based self-management interventions. Advantages to such interventions include being able to present information accessibly in simple graphics or audio-visual clips and to easily update information with the latest research available. They can also provide structured and on-going support to facilitate behaviour change, including individual assessment, goal setting, monitoring, and feedback. This support is readily available in times of need, for example, a change in medication regime or when struggling emotionally [23]. Online support groups for long term conditions can help people normalise negative emotions such as depression and boost positive emotions like hope, self-efficacy, and motivation. Additionally, sharing personal stories on line has been found to relieve social isolation, provide information in a meaningful way, and increase coping ability [24]. Computerised Cognitive Behavioural Therapy (CCBT) has also been found to reduce anxiety and depression [25]. Also, several studies have shown that diabetes specific internet-based self-management interventions can improve health behaviours, clinical outcomes, and psychological well-being [26–28].

The aim of the current study was to use a mixed-methods approach to explore the impact of using a newly developed internet-based self-management intervention called Healthy Living for People with type 2 Diabetes (HeLP-Diabetes) on the psychological well-being of adults with type 2 diabetes. The intervention was developed to target cognitions (e.g., self-efficacy, intentions, and goals); psycho-social factors (e.g., emotions such as anxiety, denial, and anger; changes in relationships); and behaviours (e.g., diet, physical activity, smoking, alcohol consumption, and taking medicines) thought to mediate clinical outcomes and health related quality of life outcomes. It was expected that using HeLP-Diabetes would therefore reduce participants' diabetes-related distress and anxiety and help participants to gain a greater sense of control and self-efficacy over their diabetes.

## 2. Methods

### 2.1. Study Design

This was a longitudinal cohort study using qualitative and quantitative methods. The quantitative aspect involved a pretest, posttest, and uncontrolled design, with data collected at baseline and 6-week follow-up via online questionnaires. The primary outcome was diabetes-related distress. Secondary outcomes were emotional distress and self-efficacy. Semistructured interviews were conducted at the same two data collection points.

### 2.2. Participant Recruitment and Procedure

Adults with type 2 diabetes were recruited from three general practices in London. Two practices were identified by the North Central London Research Consortium (NoCLor) and one through MH's previous professional relationship with them. The eligibility inclusion criteria for participants were (1) aged 18 or over, (2) diagnosis of type 2 diabetes, and (3) ability to access the internet. The exclusion criteria were participants who (1) were unable to provide informed consent (e.g., due to psychosis, dementia, severe learning difficulties); (2) were terminally ill with less than 12 months life expectancy; (3) were unable to use a computer or mobile phone due to severe mental or physical impairment; (4) had spoken or written English language skills that were insufficient to use the intervention; (5) were concurrently participating in a trial of a different self-management programme; (6) were actively suicidal or severely depressed (score above 11 on HAD-D scale); and (7) were receiving psychological therapy or counselling at the same time as the study. Ethical approval was obtained from the National Research Ethics Committee North West, Greater Manchester North, UK.

Eligible participants were identified by a GP at each practice and with the participants' permission either referred to the researcher or sent a postal invitation to take part in the study where participants contacted the researcher if they would like to take part. From this 19 participants (6 women and 13 men) were recruited (6 from referral, 13 from postal invitations). They were invited to a baseline facilitation appointment with the researcher at the practice. At this appointment participants were taken through the participant information sheet and had the opportunity to ask any questions before signing a consent form. Participants who consented were registered on the HeLP-Diabetes website and shown how to log-on using their username and password. The researcher demonstrated different parts of the programme and suggested areas they may wish to focus on based on their self-management and emotional needs. This was followed by a 30-minute semistructured interview to find out the participants' current difficulties with their diabetes and what they would like to get out of the HeLP-Diabetes website. At the end of the interview participants were emailed a link to a number of online questionnaires assessing the outcome measures. They were provided with a unique identification number to enter into the questionnaires and asked to complete this as soon as possible when they got home. Four of the participants asked to complete the questionnaire within the facilitation appointment. Participants were invited to attend a follow-up appointment after using the HeLP-Diabetes website for 6 weeks where they were given a 30-minute semistructured interview which explored whether the website had made any difference to the participants' psychological well-being and which parts of the website they found helpful or unhelpful. Afterwards they were emailed a link to complete the same set of online questionnaires as completed at baseline. The majority of participants completed these at their general practice rather than at home. During this time the researcher sat on the other side of the room, unable to see the computer screen in order to avoid experimenter bias.

The majority of patients (86%) were well-educated, having continued in education beyond A-levels, males (68%) and had been diagnosed with diabetes for more than 5 years (74%). Less than half identified as White British (42%). The ages of the participants ranged from 41 to 83 years, with a mean of 63.5 (SD = 10.7) years of age. Group and individual demographic, clinical, and self-rated previous computer experience can be found in Tables 1 and 2.

### 2.3. Intervention

HeLP-Diabetes is an internet-based self-management intervention that was developed over the course of 2 years (2011 to 2013) as part of a National Institute Health Research (NIHR) programme grant held by EM at University College London. The design of this complex intervention has been informed by theory and the needs and preferences of patients and health professionals [29]. It has been developed using a process of participatory design with users (people with T2DM and health professionals) heavily involved in the conception and creation. HeLP-Diabetes takes a holistic view of self-management and addresses a wide range of patient needs including education, lifestyle changes, medicine management, emotional management, social support with forums, and personal stories and also addresses how patients interact and work with health professionals. The components of the intervention are described in more detail in Table 3. The information provided on HeLP-Diabetes is based on NICE guidelines. Participants were asked to use the intervention at least once or twice a week for six weeks.

Participants all attended a facilitation appointment (see above for details) and were given a printed guide to using HeLP-Diabetes at home. Both were designed to improve user engagement with the intervention. They were also given the option of receiving weekly phone calls, texts, or e-mails from MH to remind them to use the website and all participants accepted this offer. On registering on the website they were also automatically signed up to a weekly HeLP-Diabetes e-mail, which encouraged use of various aspects of the intervention.

### 2.4. Data Collection

Participants were asked to complete quantitative outcome measures (diabetes-related distress, emotional distress, and self-efficacy) online through a system called Opinio [30] at baseline and at a six week follow-up. Qualitative outcome data was collected using semistructured interviews at baseline and 6-week follow-up. The interviews were audio recorded with participant's permission and transcribed verbatim.

### 2.5. Outcome Measures

#### 2.5.1. Diabetes-Related Distress

The primary outcome was diabetes-related distress measured by the Problem Areas in Diabetes Scale (PAID [10]). The PAID has 20 items focusing on areas that cause difficulty for people living with diabetes, including social situations, food, friends and family, diabetes treatment, emotions, relationships with health care professionals, and social support. An example item is “worrying about low blood sugar reactions.” Each item is scored from 0 = “not a problem” to 4 = “serious problem.” The scores are added up and multiplied by 1.25 to generate a score between 0 and 100, with higher levels indicating elevated emotional distress. A cut-off of 40 has been recommended to indicate significant levels of distress [31, 32]. The PAID has been widely used to evaluate alternate self-management interventions for people with type 2 diabetes [33], including internet-based self-management programmes [15]. It is an easy-to-administer instrument with high internal consistency [10], good validity, and responsiveness to change [34].

#### 2.5.2. Emotional Distress and Self-Efficacy

Secondary outcomes included emotional distress evaluated by a 14-item Hospital Anxiety and Depression Scale (HADS [35]) where a lower score indicates less emotional distress and self-efficacy measured by the 15-item Diabetes Management Self-Efficacy Scale UK (DMSES UK [36]) where a higher score indicates higher self-efficacy.

#### 2.5.3. Qualitative Thematic Analysis of Semistructured before and after Interviews

We developed interview guides to reflect the aims of the study. The interview at baseline asked questions about the participant's current difficulties with their diabetes and what they would like to get from the HeLP-Diabetes intervention. The interview at 6-week follow-up asked questions about whether the intervention had made a difference to participant's wellbeing and which parts of the intervention they found helpful or unhelpful.

#### 2.5.4. Usage

Use of the intervention was defined as the number of logins to the site per participant, measured by google analytics. Participants were also asked to estimate how often they had logged in over the 6 weeks at the follow-up appointment.

### 2.6. Data Analysis

#### 2.6.1. Sample Size Calculation

The primary focus of the study was on detailing the psychological changes that occurred as a result of using the HeLP-Diabetes programme, using both quantitative and qualitative methods. Using G^*∗*^Power [37, 38], it was calculated that a sample of 16, with an alpha of 0.05, would give 80% power to detect an effect size of *d* = 0.75. A sample size of 24 would give 80% power to detect an effect of 0.6, which is approximately the minimum effect size needed to be clinically useful.

#### 2.6.2. Quantitative

Descriptive analyses were performed to describe the baseline characteristics of participant's psychological well-being. The data were screened to check whether normality assumptions were met. Both PAID and HADS scores showed a deviation from normality (Shapiro-Wilk Test < 0.05); therefore the differences between pre- and postoutcome measures were analysed using nonparametric Wilcoxon signed rank tests.

#### 2.6.3. Qualitative

Data were analysed thematically. Text from the transcribed interviews with participants was coded (by key terms and phrases) and sorted by theme (domains themes and subthemes). Two experienced researchers examined the initial coding from randomly selected data independently, comparing the codes to preliminary themes, and also audited the structure of the themes [39, 40]. NVivo version 10 software [41] was used to facilitate the coding and sorting process. A summary of the results was emailed to participants for respondent validation. Four participants responded and expressed that they felt the results were an accurate reflection of their experience.

#### 2.6.4. Researchers' Background

Making the researcher values and beliefs clear is necessary to establish a basis for validity in qualitative research [39, 40]. MH has type 1 diabetes and decided not to disclose this to any of the participants in case it may have impacted on the participants' willingness to talk honestly about their feelings towards their own diabetes. CD and EM were developers of the intervention, and MH and CB were not.

## 3. Results

### 3.1. Quantitative Results

#### 3.1.1. Baseline Characteristics

The baseline PAID scores indicated that the sample had low levels of diabetes-related distress on entering the study (see Table 4). The scores on the HADS and DMSES indicated that overall the sample did not have clinical levels of depression or anxiety and they had a reasonable level of self-efficacy regarding their diabetes management.

#### 3.1.2. Before and after Comparisons

Table 4 displays the results from the pre- and postintervention data comparisons. Wilcoxon signed-rank tests showed a significant difference between participants scores on the PAID at baseline and 6-week follow-up (*Z* = 2.04, *p* = 0.04, *d* = 0.28). There was a decrease of approximately 6 points on the PAID scale, with a small to moderate effect size, indicating a reduction in diabetes-related distress. There was no significant difference between before and after scores of the HADS (*Z* = 0.89, *p* = 0.38, and *d* = 0.04) or the DMSES (*Z* = 1.87, *p* = 0.06, and *d* = 0.51) suggesting no significant changes in emotional distress or diabetes-related self-efficacy.

#### 3.1.3. Usage Data

The number of logins to the intervention recorded by google analytics over the 6 weeks ranged from 1 to 20 logins with a mean of 5.41 (S.D = 4.69). These data were somewhat supported by the self-report data. Eleven participants reported using the website more than once a week; seven reported using it less than once a week.

### 3.2. Qualitative Results

#### 3.2.1. Baseline Interviews

The qualitative data from the baseline interviews were organised into two domains: “(1) Difficulties of living with diabetes” and “(2) Hopes for HeLP-Diabetes.” The participants provided a large amount of detail on their experiences of living with diabetes and the daily difficulties that they faced. These difficulties are summarised in Table 5, as they are described in more depth in other literatures [42]. The “Hopes for HeLP-Diabetes” domain was divided into four main themes: “Changing diet and losing weight,” “Help with moods,” “Learning from other people with diabetes,” and “Wanting to learn more about diabetes.” The participants are described by their identification number and further details can be found in Table 2. Additional quotes illustrative of each theme are provided in Table 5.

#### 3.2.2. Hopes for HeLP-Diabetes

*Theme 2.1: Changing Diet and Losing Weight*. The majority of participants expressed that they would like the website to assist them in changing their diet and eating habits in order to lose weight. One participant said that she would like to know “*how to control my diet, what the right things to eat are, what to leave out, what to look for in food*” (P5). Others felt they needed help with “*willpower*” (P9) and to “*be more aware of losing weight*” (P16). Several participants spoke about wanting to lose weight through doing more exercise. For many the difficulty with exercise was being able to find enough time to do it consistently. Others wanted to know “*what sort of exercises [they could] be doing*” so they could lose weight. 

*Theme 2.2: Help with Moods*. Participants spoke about wanting to feel a shift in their emotions, approach, or attitude. Some participants expressed that they hoped the website could help them to feel more motivated. Other people wanted help with feelings such as anxiety, detachedness, mood swings, irritability, apathy, and low moods. 

*Theme 2.3: Learning from Other People*. One participant expressed that they would like “*practical tips from people who have been there, done that*” (P5). Participants commented on the benefit they felt they could gain from receiving tips and advice from other people living with type 2 diabetes. Several also felt it would be helpful to read about other people going through similar experiences as them so they would feel less isolated. 

*Theme 2.4: Learning More about Diabetes*. Participants referred mainly to the general information about diabetes they could take from the website. By gaining more information, the participants hoped to be more aware of what to expect from their diabetes and thereby have a better understanding of how to manage it.

### 3.3. Postintervention Interview Data

Following approximately six weeks of using the HeLP-Diabetes website, eighteen (of the nineteen) participants attended a follow-up interview. The postintervention interview data was organised into three domains (positive outcomes: psychological; positive outcomes: behavioural; negative experiences of the website), each containing several themes as follows. The participants are described by their identification number and further details can be found in Table 2.

#### 3.3.1. Positive Outcomes: Psychological

*Theme 1.1: Feeling Better Informed and More Aware*. Participants reported feeling that the website had offered them new information regarding their diabetes or information they had previously learnt but felt it was beneficial to be reminded of. This new or updated information seemed to help the participants in different ways. For some it helped them gain a better understanding of their symptoms such as fatigue: “*…I really did not know that being constantly exhausted was part of diabetes…*” (P4). Others felt that the new information had the potential to change their current behaviours in relation to their diabetes and therefore help them to gain more control over their condition.

*It's broadened my mind about everything. So, it's opened things up to me that I wouldn't have… if I'd have just gone on in my own little way, I would still be doing the same things so it has changed me, definitely, and I hope for the better. (P12)*

Participants talked about the benefit of being able to refer back to the information on the website in times of need. This provided a level of comfort in knowing that the information and support was readily available to them. It also allowed them to read and digest the information at their own pace or to refer back to it if they had forgotten something. One participant felt this was helpful as it allowed him to be less reliant on his GP and the NHS.

*It's brilliant, it's great because it means that if I have a particular concern or if I feel I'm going off track in any way, in any aspect of my living then I can refer back to that and it would, you know, on present experience it would probably give me the answer or point me in the right direction to an answer and make me less reliant on a GP appointment. And so that's giving me instant input and giving the NHS less time to have to spend on concerns that can be answered in there. (P5)*

No participants reported using any other self-management interventions during the course of the study. 

*Theme 1.2: Taking Diabetes More Seriously*. Participants reported feeling more aware of the “*dangers*” (P2) of diabetes and consequently felt more motivated to improve their self-management. Participants did not report negative emotions resulting from the information about potential complications, possibly because it was presented along with information about how to avoid these complications.

*I suppose when you go into the risks and stuff about your body and different things that can happen but the main message that's coming across is this is manageable, you can manage it, here are some things to do it, you know, why shouldn't you manage it? (P7)*

Participants spoke about experiencing a shift in perception with regards to their diabetes. They talked about having previously viewed diabetes “*a bit casually*” (P4) but in reading more about diabetes complications they felt they were taking their condition more seriously. For several participants, taking their condition more seriously came with an increased sense of responsibility and ownership over managing their condition. 

*Theme 1.3: Increased Self-Efficacy and Support*. Participants reported feeling an increased sense of self-efficacy in managing their diabetes following using the website.

*I'm more aware of what is going on. I feel, like, I'm in control in a way. (P1)*

In particular participants reported an increase in motivation and self-efficacy through seeing other people on the website who were managing their diabetes.

*You know, the people are just like me and they're getting on with it… And they're doing it at their age or whatever and there's no excuse for me not to do it. (P7)*

As well as boosting self-efficacy, social comparison seemed to help the participants to feel less isolated in their experiences and helped them to normalise their feelings around diabetes self-management.

*People often feel guilty about slipping up with their diet by indulging in something that they were trying not to eat, or putting off going for that swim they had planned; it was very useful to read that. (P9)*

Several participants spoke about the social support they took from the website. This seemed to help alleviate a general sense of isolation as well as providing a source of answers and information that they may not have felt was readily accessible elsewhere. One participant described that the people on the website now felt “*part of your support community*” (P7).

Additionally, participants described feeling that it was beneficial to have advice from medical professionals available when needed. This again may have helped to alleviate a sense of loneliness and uncertainty with regards to their condition.

*I felt it was as though I was, sort of, face to face with a practitioner to the extent that that's what they would tell me if I explained a certain symptom or a certain problem related to diabetes with them. (P5)*

*Theme 1.4: Improved Management of Worries and Low Mood*. Participants spoke about taking a new approach to managing their worries and low mood since using the website. Several participants seemed to find a new determination to acknowledge that “*life goes on*” (P1) despite diabetes. They described feeling better able to manage their moods. This seemed mainly to come through finding an alternative way of thinking about their situation and trying to accept day to day worries and only act on them when necessary for managing their condition.

*I've just accepted I'm a diabetic, and I've just got to live with it, so I do not, sort of, get my knickers in a knot about it; the only time I sort of worry about it is when I start to feel faint or nauseous or something like that, then I check to see what my sugar is like. (P8)*

#### 3.3.2. Positive Outcomes: Behavioural

All but four participants reported some aspect of behavioural shift following the use of the website. The behaviours reported changes to eating habits; changes to exercise; and other changes to self-management. 

*Theme 2.1: Changes to Eating Habits*. Several participants started eating more fruits and vegetables.

*Looking at it, it's made me realise I have to change things. And I knew I had to change things, but not really how, but that has helped me to see, and one of the things I'm doing since is I'm getting a lot of fruit in. So I just leave the fruit lying around, whereas normally, what I would have done is just have some fruit when I felt like getting it. (P19)*

Other people reported becoming more aware of portion sizes, snacking, and managing their intake of sugary foods.

*I am much more conscious also of not snacking in between. (P15)*

*Theme 2.2: Changes to Exercise*. Participants reported that the website had highlighted to them “*that exercise is as important as anything*” (P3), with regards to their diabetes self-management. A couple of participants spoke about using advice from the website to introduce exercise into their day-to-day lives, whether it was walking a bit further than usual or dancing while doing the housework. For some participants, the website prompted them to do more exercise, on top of what they were already doing.

*And even when I do water aerobics, I used to come home so tired and then I just wait again for the next Tuesday but now I try and do some every day. (P2)*

*Theme 2.3: Other Changes to Self-Management*. Several participants spoke about changes they had made to the medical management of their diabetes. These participants reported that the website had prompted them to check their blood sugar levels more frequently.

*Because the more I thought about it the more I could, for example, take my readings and control my blood sugar. (P3)*

Other changes included learning more about managing hypos and how to treat them, as well as being more careful about taking medication with food.

*Because I did have hypoglycaemia twice, to 3.1 and it was very interesting what they told me, what to do, just in case it happens…  I have always with me sugar cubes in my bag, but I did not know how many to eat, for instance. (P15)*

#### 3.3.3. Negative Experiences of the Website

*Theme 3.1: Information Not New or Helpful*. More than half of the participants expressed disappointment in finding that the information on the website did not meet their needs. It was either information they already knew (and did not need refreshing) or that was not helpful to them.

*It probably didn't give me so much information as I might have hoped. (P3)*

Several participants spoke of this in relation to the areas of the website that aimed to help people improve their moods. These participants reported that they did not experience difficulties with their moods and therefore did not find this section of the website to be of use to them.

*I didn't find it particularly helpful because I just thought… it's about if you get depressed but it said that people with diabetes are more prone to get depression and I think, maybe because I'm lucky, that has't [sic] happened to me. (P12)*

*Theme 3.2: Not Feeling Able to Relate to the Experiences of Others*. Participants reported feeling frustrated by the views and experiences of others on the website which did not fit with their own. The coping styles of people on the website, for example, being emotionally expressive, may have opposed the strategies that certain participants had adopted to help them cope with their diabetes, for example, avoidance.

*No, they had sort of little stories about people feeling so distraught when they first heard they had diabetes, and I thought, oh, silly people – that's all I thought…. No, I couldn't relate to them whinging, no, no. (P4)*

*Theme 3.3: No Changes to Certain Aspects of Diabetes-Related Behaviour. *Participants spoke of aspects of diabetes self-management that they had not been able to change. They reported intentions to change their behaviour based on the information they had read on the website. However, the difficulty remained in following the intention with action to change their behaviour.

*Well as I say it hasn't had a practical impact on me yet because I haven't organised myself to adopt some of the things I have read and thought were very good to adopt. (P5)*

A few participants expressed shame or guilt in relation to not being able to change their behaviour relating to their diabetes. However, these emotions did not seem to motivate change and therefore may have caught the participants in a vicious cycle of being self-critical and unmotivated and then more self-critical.

*I ought to do something a bit more than I am doing, made me feel, perhaps, even… I'm very good at feeling guilty these days. (P6)*

A couple of participants expressed that they felt they would need something more than the website to motivate them to change their behaviour, namely, diabetes-related complications or more in-depth professional input.

*I haven't really changed anything that I should or shouldn't be doing. Maybe, like I said, when [a complication] happens to me then I might start thinking a bit more about it, but so far, like I said, touch wood, nothing serious yet. (P10)*

*Theme 3.4: Technical Frustrations*. The website did cause some participants to feel anger and frustration when using it. This happened mainly in relation to when the website did not work as hoped or did not live up to expectations. This may have therefore led to the participant withdrawing from using the site due to the negative association.

*Why it didn't feel intuitive? Well, I intuitively did what I would normally do, and it didn't give me the answers, and so I just sort of thought, oh well, to hell with it. (P14)*

*Theme 3.5: Feeling Guilty about Not Using the Website*. Participants expressed feeling guilt in relation to not using the website in accordance with what was asked of them for the study. This guilt was accentuated by weekly emails and phone calls from the researcher to remind them to use the site and to check in with their progress.

*I think the only difference I could honestly say it made was that I knew it was there and that I felt guilty about it, really. (P11)*

Occasionally this guilt and frustration towards not being able to use the website seemed to be turned inwards and resulted in self-negative feelings.

*Inadequate feelings, you know. Oh, *C*^*∗∗∗∗*^t, you know, I can't even remember the passwords – that sort of thing. (P17)*

### 3.4. Comparison of before and after Themes

Comparison of the themes from the preintervention and the postintervention interviews shows that participants' expectations for the site were generally satisfied. Their main preintervention hopes were to improve their management of diet and moods; these improvements were reflected in the postintervention themes. They also wished to learn from others' experience of diabetes. There is a suggestion that this did not always work well, as one of the negative outcomes was that some participants did not feel able to relate to the experiences of other patients (Table 6).

## 4. Discussion

The aim of the study was to use a mixed-methods approach to examine the impact of an internet-based self-management intervention (HeLP-Diabetes) on the psychological well-being of people with type 2 diabetes over a 6-week period. The quantitative results showed a significant decrease in diabetes-related distress following access to the intervention with no significant change in levels of anxiety or depression. There was no significant change in diabetes-related self-efficacy despite this measure showing an average increase of 12 points.

The qualitative data expanded and explained the quantitative findings: participants reported that having access to practical information made them more aware of why and how they could self-manage. They stated they felt more supported and more able to manage low mood, as well as making improvements to their eating habits, exercise routine, and medical management. Negative impacts associated with using the intervention were also found, in the form of guilt (about not using the website, or about not making the desired behavioural changes), and frustration with navigating the website.

The above quantitative findings confirm a recent Cochrane review of computer-based diabetes self-management interventions for adults with type 2 diabetes [43] which showed overall no impact on clinical depression scores. They contrast with previous research [18] that has shown a diabetes education and self-management programme to have no effect on diabetes-related distress as measured by the PAID. This may possibly reflect the strong focus that HeLP-Diabetes has on emotional management and role management by including a cCBT module, forums, and personal stories. The qualitative findings support data showing that internet-based diabetes self-management interventions can lead to small significant increases in self-efficacy [27, 44] and changes to eating habits and exercise.

Negative experiences associated with using the intervention included feelings of guilt for not using the website as suggested or making any behavioral changes. This emotional reaction may link with the “Anger and self-criticism” that was described in the preintervention interviews. Within this latter subtheme, participants described their guilt and self-annoyance in not being able to make the “correct” decisions in their day-to-day lives or do all the things that were expected of them from their health professionals, for example, eat food with low sugar content. This finding is consistent with other qualitative research which found that patients self-attributed blame for being unable to achieve treatment goals. Furthermore this study reported that patients frequently expressed frustration and disappointment inwardly through self-deprecating comments [45]. It is possible that these feelings of self-blame and self-criticism around their self-management extended to their difficulties in logging on to the website as agreed and thus reinforced their critical self-perception. The likelihood of this emotional impact occurring may be greater for older adults with type 2 diabetes who may find the website technologically challenging or for adults of working age who cannot find the time to dedicate to the website. With the amount of demands that are placed on people with type 2 diabetes to self-manage their condition, it may be for some people that using a website is one demand too many.

Although generalisations from qualitative research should be made with caution, it is important to consider how the results of the current study may or may not extend to other users of the HeLP-Diabetes website. Aspects of the methodology that may have limited the extent to which the sample was representative of the larger population included the sample size, the characteristics of the sample, demand characteristics, and length of intervention. In particular, the sample overall was well educated, mostly computer literate and with low levels of distress. These characteristics may reduce the transferability of the findings.

A particular strength of this study was the use of a mixed methodology. The qualitative interviews allowed for exploration of the participants' complex views of their diabetes and the emotional impact of the HeLP-Diabetes programme. The interviews allowed the participants to have more flexibility and give more detail in their responses to questions. A quantitative approach also had the advantage of allowing a more accurate comparison of responses obtained before and after the intervention. The two methods were therefore used in a complementary fashion to gather both detailed views and more precisely and potentially more subtle changes in emotional and cognitive constructs.

This study also highlights some important factors for GPs and Practice Nurses to consider when deciding who the website might be more or less helpful for. The results showed that 47% of participants had not previously received any structured education around their diabetes. For people who do not have the time or ability to attend a face-to-face course, HeLP-Diabetes can provide an accessible alternative to receiving important diabetes-related information. However, patients who are not familiar or comfortable with online resources or patients who are already dealing with many demands in their day-to-day lives might find it challenging to make best use of the website. If these patients already have a tendency to be self-critical regarding their difficulties with diabetes self-management, then it is possible that this intervention may reinforce their sense of “failure” if they are unable to use the website. Conversely, it might be that HeLP-Diabetes could be particularly helpful for people who are newly diagnosed. This unfortunately was not established in the current study as there was only one newly diagnosed participant. However, the majority of participants expressed that they felt the website would be most helpful to people who had just received their diagnosis. Research has shown that it is commonly assumed that patients with a new diagnosis have difficulty in retaining information due to the resulting shock and stress [46]. However, a qualitative study involving 40 newly diagnosed patients with type 2 diabetes found that most patients wanted more information about diabetes management at the time of diagnosis [47].

## 5. Conclusions

The findings of the current study further highlight the need for health professionals to consider the psychological impact of living with diabetes and to take steps to help their patients address it. These preliminary findings demonstrated that the use of HeLP-Diabetes was associated with a reduction in diabetes-related distress. The HeLP-Diabetes website therefore provides a viable option to GPs and Practice Nurses for helping their patients increase their awareness of their condition; appreciate the seriousness of their diabetes whilst increasing their self-efficacy in managing it; and learn from others with type 2 diabetes so that they feel better able to manage their anxieties and low moods. The findings need to be replicated with a larger sample size and more robust design. This research is currently underway [48]. If confirmed, this would support the use of internet-based self-management support such as HeLP-Diabetes, particularly as high levels of distress appear to be causally related to poor glycaemic control [8, 10], and reducing distress may help improve control and reduce the risk of diabetes-related complications [12–15].

## Figures and Tables

**Table 1:** Demographic and clinical characteristics of participants.

Variable	Mean (SD) or frequency (%)
*Demographics *	
Age (years)	63.5 (10.7), range 41–83
Gender	32% female
	68% male
Ethnicity	
White British	8 (42%)
White Irish	3 (16%)
White other	2 (11%)
Bangladeshi	2 (11%)
African	1 (5%)
Caribbean	1 (5%)
Chinese	1 (5%)
Other Asian	1 (5%)
Highest level qualification	
Secondary school	1 (5%)
GCSE's	1 (5%)
A-levels	1 (5%)
Further qualifications (e.g., diploma)	7 (38%)
Undergraduate degree	2 (11%)
Postgraduate degree	7 (37%)
Marital status	
Married	9 (47%)
Single	5 (26%)
Divorced	4 (21%)
Preferred not to state	1 (5%)
First language	
English	15 (79%)
Spanish	1 (5%)
French	1 (5%)
Swahili	1 (5%)
Mandarin	1 (5%)
*Clinical *	
Duration of diabetes	
0–6 months	1 (5%)
1-2 years	1 (5%)
2–5 years	3 (16%)
5–10 years	6 (32%)
10+ years	8 (42%)
Current or previous diabetes-related complications	
Yes	8 (42%)
No	11 (58%)
*Self-rated computer experience* ^*∗*^	
Advanced	7 (37%)
Intermediate	7 (37%)
Basic	5 (26%)

^*∗*^Advanced (e.g., work is to do with the Internet); intermediate (e.g., used or currently use the Internet regularly); basic (e.g., used the Internet a few times but not often).

**Table 2:** Individual demographic information for each participant.

Participant	Gender	Age	Ethnicity	Duration of diabetes	Previous or current complications	Self-rated previous computer experience^∗^
1	Female	40s	African	10+ yrs	No	Advanced
2	Female	60s	Caribbean	0–6 months	Yes	Basic
3	Female	60s	Bangladeshi	10+ yrs	Yes	Advanced
4	Female	70s	White British	10+ yrs	Yes	Intermediate
5	Male	60s	White British	2–5 yrs	No	Intermediate
6	Male	80s	White British	10+ yrs	Yes	Basic
7	Male	40s	White British	2–5 yrs	No	Intermediate
8	Male	60s	White British	10+ yrs	Yes	Advanced
9	Male	60s	White British	2–5 yrs	No	Advanced
10	Male	60s	Other Asian background	5–10 yrs	No	Advanced
11	Female	70s	White British	1-2 yrs	No	Intermediate
12	Male	50s	White Irish	5–10 yrs	No	Basic
13	Male	40s	Bangladeshi	5–10 yrs	Yes	Advanced
14	Male	60s	White British	10+ yrs	Yes	Basic
15	Female	70s	White (other)	5–10 yrs	No	Intermediate
16	Male	70s	White (other)	10+ yrs	No	Intermediate
17	Male	70s	White Irish	5–10 yrs	No	Basic
18	Male	50s	Chinese	10+ yrs	Yes	Advanced
19	Male	60s	White Irish	5–10 yrs	No	Intermediate

^∗^Advanced (e.g., work is to do with the Internet); intermediate (e.g., used or currently use the Internet regularly); basic (e.g., used the Internet a few times but not often).

**Table 3:** HeLP-Diabetes module names and descriptions.

Module names	Descriptions
Understanding Diabetes Treating Diabetes Living and Working with Diabetes	Three modules aimed at improving role and behavioural management. Information about what diabetes is (including possible complications), how to treat it (information about different medicines and alternative treatments), and living and working with diabetes (focusing on the impact it may have on relationships).

Staying Healthy	Focused on improving behavioural management and helping people to make lifestyle changes with regards to diet, physical activity, taking medicine, reducing smoking and alcohol consumption, and working with a diabetes care team.

Forum & Help	Focused on improving emotional and role management. Includes an interactive forum and personal stories of real people with type 2 diabetes.

My Health Record	Focused on improving behavioural management. A module that can interact with the user's health professional and contains the user's personal information, care plan, a list of medicines, appointments, and self-monitoring data.

Managing my Feelings	Focused on improving emotional management. Contains a computerised cognitive behavioural therapy course called “Living life to the full,” which was adapted for people with diabetes by Williams [49]. This module aims to provide strategies to manage symptoms of anxiety and depression. It also contains information on mindfulness techniques.

News and Research	Provides the latest news articles, research trials, and advice on media coverage about type 2 diabetes.

**Table 4:** Outcome measures at baseline and 6-week follow-up.

Measure	Before M (SD)	After M (SD)	*Z* score	Wilcoxon Signed Ranks	Cohen's *d*
				Sig	
PAID	26.32 (20.88)	20.97 (16.53)	2.04	0.04^∗^	0.28
HADS	12.33 (10.15)	12.78 (11.20)	0.89	0.38	0.04
DMSES	90.67 (20.17)	102.78 (26.66)	1.87	0.06	0.51

*Notes*. PAID = Problem Areas in Diabetes scale. HADS = Hospital Anxiety and Depression scale. DMSES = Diabetes Management Self-Efficacy scale UK. Please refer to Section 2 for an explanation of direction and range of each scale. Level of significance = 0.05^∗^.

**Table 5:** Domains, themes, su-themes, and illustrative quotes for the preintervention data.

Domains, themes, and subthemes	Illustrative quotes
*(1) Difficulties of Living with Diabetes *	
(1.1) Impact on psychological well-being	
(1.11) Worries about long term complications	“I ask God, you want to take something, take a leg but let me have my eyes.” (P4)
(1.12) Concerns about medication and related side-effects	“you take medication, they treat one thing, they give you complications and the others, so… there's other things that play up in my mind as well. Knowing, okay, this is treating these, but there's side-effects as well.” (P1)
(1.13) Desire for normality	“that's part of wanting to feel as normal as possible and to feel as normal as possible could involve a degree of pushing to one side what actually one needs to do to remain stable and to manage one's condition.” (P5)
(1.14) Managing by minimising concerns	“it's not treated at a deadly serious level, it's treated lightly probably to disguise what's going on underneath.” (P14)
(1.15) Anger and self-criticism	“I could be quite bad-tempered sometimes, and possibly…it might have been caused by…the thought of the diabetes. I could lose my temper.” (P6)
(1.16) Feeling depressed and apathetic	“… I'm not a depressing type of person, but it can make you feel down sometimes.” (P17)
(1.2) Difficulties with self-management	
(1.21) Battles with eating and weight	“It's a bit tricky because I like food and I like cooking, and so it's…yes, it's quite a challenge” (P11)
(1.22) Difficulty controlling blood sugar levels	“It has taken an awful long time, not to take too much insulin and therefore get hypos and/or, not take enough and my diabetes goes up.” (P3)
(1.23) Lack of control or predictability	“There are mysteries and disconnect between the prescribed treatment and the result.” (P14)
(1.24) Difficulty sticking to a regime	“And so my main problem - apart from the odd lapses when I completely forget to take my medication, is how to stick to a regime which is going to have a positive impact on my health.” (P5)
(1.3) Social pressures and impact on social roles	
(1.31) Pressure from others	“My children are very supportive; they just said, dad, you can't have that, or they will ask at a restaurant, and now it's…got too much sugar in, you just can't have it. So, it's quite nice. Sometimes a bit of a pain in the butt” (P8)
(1.32) Impact on role in family	“It contributes to one's constant feeling of failure as a father, that you're not bringing up your child properly, but… if you can't rush out and do things.” (P7)
(1.33) Impact on work role and hobbies	“We both love going to museums and art galleries and stuff and now I can't. I cannot walk round an exhibition, I'm too tired.” (P4)
(1.34) Impact on social life and society	“Often I'm faced with big meals and lots of drink, and often you can get away with it. Often you'd find you'd be giving offence if you don't.” (P9)
*(2) Hopes for HeLP-Diabetes *	
(2.1) Changing diet and losing weight	“Hints on how I can lose weight and control my diabetes more.” (P3)
(2.2) Help with moods	“That, Managing my Feelings – that looked quite interesting. That was something that has made me feel quite happy, actually.” (P19)
(2.3) Learning from other people with diabetes	“The forum, if I go there, they have the same situation, so we can share, we can give some information, we can help each other.” (P13)
(2.4) Wanting to learn more about diabetes	“Because I want to learn more, learn what I can do, the effects of it and whatever, you know, because I don't want to be ignorant or that, I want to know about this thing and know as much as I can about it.” (P2)

**Table 6:** Outline of domains and themes for postintervention interviews.

Domains and themes	Prevalence
*(1) Positive outcomes: psychological *	
(1.1) Feeling better informed and more aware	General
(1.2) Taking diabetes more seriously	Common
(1.3) Increased self-efficacy and support	Common
(1.4) Improved management of worries and low mood	Common
*(2) Positive outcomes: behavioural *	
(2.1) Changes to eating habits	Common
(2.2) Changes to exercise	Variant
(2.3) Other changes to self-management	Variant
*(3) Negative experiences of the website *	
(3.1) Information not new or helpful	Common
(3.2) Not feeling able to relate to the experiences of others	Variant
(3.3) No changes to certain aspects of diabetes-related behaviour	Common
(3.4) Technical frustrations	Common
(3.5) Feeling guilty about not using the website	Variant

*Notes*. General = theme applies to 13–18 participants. Common = theme applies to 7–12 participants. Variant = theme applies to 4–6 participants.
